# The prevalence of elevated hemoglobin A1c in patients undergoing coronary artery bypass surgery

**DOI:** 10.1186/1749-8090-3-63

**Published:** 2008-11-24

**Authors:** Milo Engoren, Robert H Habib, Anoar Zacharias, Thomas A Schwann, Christopher J Riordan, Samuel J Durham, Aamir Shah

**Affiliations:** 1 Department of Anesthesiology, University of Toledo Health Science Campus, St. Vincent Mercy Medical Center, 2213 Cherry Street, Toledo, OH 43608, USA; 2 Cardiothoracic Surgery, University of Toledo Health Science Campus, St. Vincent Mercy Medical Center, 2213 Cherry Street, Toledo, OH 43608, USA

## Abstract

**Background:**

Diabetes mellitus has become a major health issue in the United States and contributes to morbidity and mortality from coronary artery disease. Despite lifestyle changes and medications that have been shown to decrease complications and death, many persons have poor glycemic control. The purpose of this study is to determine the prevalence of elevated Hemoglobin A1c levels, a marker of glycemic control in patients presenting for coronary artery bypass surgery, and to determine if risk factors for diabetes mellitus could identify those patients with an elevated hemoglobin A1c.

**Methods:**

All patients undergoing coronary artery bypass surgery had hemoglobin A1c levels determined immediately preoperatively. Proportions were used to describe the number of patients with elevated levels. Linear regression and receiver operator characteristic curves were used to evaluate the accuracy of risk factors to identify patients with elevated levels.

**Results:**

83 of 87 (95%) diabetic patients had elevated A1c levels (≥ 6.0%), with 55 of 87 (63%) having inadequate control – A1c levels ≥ 7.0. 93 of 163 (57%) non-diabetic patients had elevated A1c levels (≥ 6.0%), with 19 (12%) having levels ≥ 7.0%. Risk factors for diabetes mellitus poorly predicted which patient had elevated A1c levels.

**Conclusion:**

The prevalence of elevated hemoglobin levels in patients undergoing coronary artery bypass surgery is high and routine measurement should be done to permit institution of lifestyle modifications and medication changes that decrease complications and death from diabetes mellitus.

## Background

The prevalence of diabetes mellitus(DM) is increasing in the United Sates and has become a major public health issue [1]. Nearly 21 million Americans – 7% of the population – have DM, including over 6 million who are undiagnosed [1]. Additionally, there are 41 million Americans with pre-diabetes [2]. DM is a risk factor for coronary artery disease and its presence portends a worse outcome – both short and long term – in patients undergoing coronary artery bypass surgery [3,4]. Following recent studies that have shown the benefits of tight glycemic control in both diabetic and non-diabetic patients [5,6], we instituted protocols designed for tight glycemic control in both the intensive care unit and the stepdown unit. We noticed, however, that some patients without any history of DM were still requiring antiglycemic therapy upon discharge and that other patients with a history of DM remained poorly controlled despite resumption of their usual antiglycemic medicines and eating a hospital provided diabetic diet. To better help us provide for patients' post-discharge care, we began to routinely check hemoglobin A1c (HbA1c). HbA1c is the glycosolated form of hemoglobin and its level is proportional to the average glucose level over the past 2–3 months [7]. While not recommended for the diagnosis of DM [8], specific HbA1c levels are recommended as treatment goals by national organizations [9,10] and we used these levels in guiding post-discharge therapy and instructions. The purpose of this study was to determine the prevalence of elevated HbA1c in the diabetic and non-diabetic subcohorts of a cardiac surgery population and to determine if age, height, weight, and body mass index – standard risk factors for DM could be used to predict elevated HbA1c levels.

## Methods

This retrospective study was approved on August 30, 2007 by the St. Vincent Mercy Medical Center Review Board, which waived informed consent. HbA1c levels had been drawn immediately prior to surgery in all patients undergoing cardiac surgery as part of their routine care and the results included in our computerized database. Data are presented as histograms, proportions, and means ± standard deviation and analyzed with student t test and Chi square test. We separately used linear regression on patients with and without DM to predict HbA1c levels based on age, height, weight, and body mass index. Then we used receiver operator characteristic curves to measure the predictive accuracy of the two linear regressions. The results are given as area under the receiver operator characteristic curve (c-statistic ± standard error). SPSS 13.0 (SPSS, Inc., Chicago, IL) was used for data analysis. The power analysis was based on determining the 95% confidence interval for the point estimate within 5% of the true proportion of patients with elevated HbA1c – defined as ≥ 6.0% – assuming the true proportion was 0.2. This required 250 patients [11].

## Results

Data were collected from 250 consecutive patients undergoing CABG (January 2007 – July 2007) in whom preoperative HbA1c was measured. Of these patients, 212 underwent isolated CABG surgery and the other 38 CABG combined with carotid, valve, or aortic surgery. The proportion of patients with DM was similar in the two groups. Patients were 65 ± 11 years of age, weighed 88 ± 20 kg, were 172 ± 20 cm tall, and had body mass index (BMI) 30 ± 6. One hundred seventy seven (71%) of the patients were male and 87 (35%) patients had diabetes mellitus – 13 were receiving insulin at home, 59 oral hypoglycemic agents, 5 both, and 10 neither.

**Figure 1:**
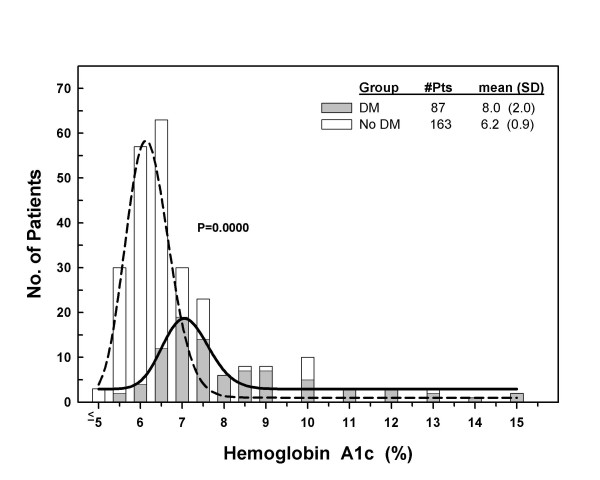
**Histogram showing the distribution of hemoglobin A1c levels in patients with and without diabetes mellitus undergoing CABG surgery**. The dashed curve (patients without diabetes mellitus) and solid curve (patients with diabetes mellitus) show the expected normal distribution for these populations.

The distribution of HbA1c values are shown for the diabetics and non-diabetics in Figure 1. Diabetic patients had higher levels (8.0 ± 2.0% v. 6.2 ± 0.9%, p < .001) than non-diabetic patients. Sixty four percent (95% confidence interval = 58–72%) of the patients had A1c ≥ 6.0%. Only four (5%) of the diabetic patients had HbA1c in the normal range (HbA1c < 6.0%) and another 28 (32%) had HbA1c levels that met the ADA goal of < 7.0%. The remaining 55 (63%) had unacceptably high levels, with 12 (14%) having levels ≥ 10%. Over half (n = 93, 57%) of the patients without a history of diabetes mellitus had elevated HbA1c ≥ 6.0% with 19 (12%) of them having HbA1c ≥ 7.0%. In patients with DM, the standard risk factors of age, height, weight, and body mass index poorly predicted elevated HbA1c: c-statistic = .639 ± .064, p = .027 for HbA1c ≥ 7.0% and c-statistic = .470 ± .204, p = .839 for HbA1c ≥ 6.0%. In non-diabetic patients, results were c-statistic = .742 ± .048, p < .001 for HbA1c ≥ 7.0% and c-statistic = .615 ± .045, p = .013 for HbA1c ≥ 6.0%.

## Discussion

We found that many patients with a history of diabetes mellitus undergoing CABG had elevated HbA1c levels suggesting poor glycemic control. Additionally, we found that over half of the non-diabetic patients undergoing CABG had elevated HbA1c levels suggestive of prediabetes or diabetes mellitus. Standard risk factors of age, height, weight, and body mass index were inadequate predictors of elevated HbA1c and should not be used instead of HbA1c as a screening test in CABG patients.

One previous study has evaluated A1c levels in diabetic patients undergoing CABG. Cohen et al. found that A1c levels were 7.03 ± 1.50% and were associated with increased risk of complications in an Israeli population [4]. They did not evaluate non-diabetic patients. We found higher A1c levels (8.0 ± 2.0%) in our diabetic population. Further study is needed to determine the reasons for the poorer control in Ohio patients than in Israeli patients. A study in non-diabetic patients undergoing percutaneous coronary interventions (PCI) found a 30% prevalence of elevated (≥ 6.0%) A1c levels and another 3% had A1c levels ≥ 7.0% and that levels ≥ 6.% were related to a worse 12 month outcome [12]. Our higher rate (57% ≥ 6.0% and 12% ≥ 7.0%)of abnormal A1c levels may indicate more extensive disease that would necessitate CABG instead of PCI. Kowalska et al. found that A1c levels were positively associated with number of diseased vessels [13].

DM is associated with organ damage to the nerves, eyes, kidneys, blood vessels, and heart leading to morbidity, decreased quality of life, and increased mortality [9]. While a prospective, randomized trial of tight glycemic control in patients with type 1 DM has shown a 57% risk reduction in cardiovascular events over a mean followup of 17 years [14], long-term prospective studies evaluating tight control in type II DM have not shown such impressive results. However, the UK Prospective Diabetes Study Group has shown that intensive control with antiglycemic agents compared to conventional therapy is associated with a lowering of myocardial infarction risk that is of "borderline significance" of 14% and that the use of metformin is associated with a lower risk of death in overweight diabetic patients [15,16]. Improved glycemic control regardless of method was associated with a lowered risk of myocardial infarction [17]. A recent meta-analysis found that each 1% lowering of HBA1c was associated with a 18% reduction in relative risk of developing cardiovascular disease [18].

In a nationwide survey only half of patients with DM met American Diabetes Association clinical practice recommendation of HbA1c < 7% and 30% had HbA1c ≥ 8% [19]. Studies have shown that lifestyle modification, such as weight loss, dietary changes, and exercise decease the likelihood of developing DM [20,21]. Although cardiac surgery is a major life stressor it is also an opportunity for health care professionals to intervene and educate patients and to institute therapy for secondary prevention of disease. Smoking cessation and low-fat diets are common lifestyle interventions in patients undergoing cardiac surgery. Antihypertensives, lipid lowering drugs, such as statins, and aspirin are commonly started or adjusted for better control in patients who have undergone CABG.

While the association with glucose intolerance and coronary artery disease is well described [22], our study is the first to show the high prevalence in patients undergoing CABG of undiagnosed prediabetes or diabetes mellitus and poorly controlled glycemia in patients with known diabetes mellitus. Given the benefits that may be achieved by tighter glycemic control [15-18,20,21], screening of all patients undergoing CABG is recommended. Studies are needed to determine if CABG surgery should be delayed to lower the A1c level and to determine the level to which it should be lowered.

While not all patients with elevated HbA1c have DM – the diagnosis should be made on the basis of fasting glucose levels and glucose tolerance test, they are all, at least, at increased risk for developing DM. At the minimum, we recommend lifestyle changes of weight loss, diet changes, and exercise. All patients with elevated HbA1c should followup with their primary care physician or endocrinologist for further diagnostic evaluation. For patients still requiring insulin to control hyperglycemia at time of hospital discharge, we recommend discharge with an appropriate individualized antihyperglycemic medicine. Diabetic patients with an elevated HbA1c are also given similar counseling and adjustments made, if necessary, in their usual diabetic medications.

The limitation of this study is that it was conducted at one hospital, which may limit its generalizabilty. Patients in other areas of the country may have different prevalences of DM and risk factors for DM and this study needs to be validated in other geographical regions. The prevalence of DM in Ohio, 7.5%, is similar to the prevalence (7.3%) in the United States, which suggests that our findings of elevated HbA1c in 57% of nondiabetic patients undergoing CABG would hold true elsewhere in the United States [23].

In conclusion, we found that 57% of non-diabetic patients undergoing CABG had elevated HbA1c. Additionally, 96% of diabetic patients undergoing CABG had elevated HbA1c. These high prevalences of elevated HbA1c suggest routine evaluation in all adult patients undergoing CABG in order to institute lifestyle and medication changes that improve glycemic control.

## Competing interests

The authors declare that they have no competing interests.

## Authors' contributions

ME conceived the study. ME, AZ, TS, CR, SD, AS participated in data collection. ME, RH drafted the manuscript. All authors revised it critically for important intellectual content and have given final approval of the version to be published.
